# Increased occurrence of protein kinase CK2 in astrocytes in Alzheimer’s disease pathology

**DOI:** 10.1186/s12974-015-0470-x

**Published:** 2016-01-06

**Authors:** Andrea F. N. Rosenberger, Tjado H. J. Morrema, Wouter H. Gerritsen, Elise S. van Haastert, Hripsime Snkhchyan, Riet Hilhorst, Annemieke J. M. Rozemuller, Philip Scheltens, Saskia M. van der Vies, Jeroen J. M. Hoozemans

**Affiliations:** Alzheimer center & Department of Neurology, Neuroscience Campus Amsterdam, VU University Medical Center, De Boelelaan 1118, 1081 HZ Amsterdam, The Netherlands; Department of Pathology, Neuroscience Campus Amsterdam, VU University Medical Center, De Boelelaan 1117, 1081 HV Amsterdam, The Netherlands; PamGene International BV, Wolvenhoek 10, 5211 HH ‘s-Hertogenbosch, The Netherlands

**Keywords:** Alzheimer’s disease, Casein kinase 2, Protein kinase CK2, Neuroinflammation, Astrocytes

## Abstract

**Background:**

Alzheimer’s disease (AD) is the most common neurodegenerative disease. In addition to the occurrence of amyloid deposits and widespread tau pathology, AD is associated with a neuroinflammatory response characterized by the activation of microglia and astrocytes. Protein kinase 2 (CK2, former casein kinase II) is involved in a wide variety of cellular processes. Previous studies on CK2 in AD showed controversial results, and the involvement of CK2 in neuroinflammation in AD remains elusive.

**Methods:**

In this study, we used immunohistochemical and immunofluorescent staining methods to investigate the localization of CK2 in the hippocampus and temporal cortex of patients with AD and non-demented controls. We compared protein levels with Western blotting analysis, and we investigated CK2 activity in human U373 astrocytoma cells and human primary adult astrocytes stimulated with IL-1β or TNF-α.

**Results:**

We report increased levels of CK2 in the hippocampus and temporal cortex of AD patients compared to non-demented controls. Immunohistochemical analysis shows CK2 immunoreactivity in astrocytes in AD and control cases. In AD, the presence of CK2 immunoreactive astrocytes is increased. CK2 immunopositive astrocytes are associated with amyloid deposits, suggesting an involvement of CK2 in the neuroinflammatory response. In U373 cells and human primary astrocytes, the selective CK2 inhibitor CX-4945 shows a dose-dependent reduction of the IL-1β or TNF-α induced MCP-1 and IL-6 secretion.

**Conclusions:**

This data suggests that CK2 in astrocytes is involved in the neuroinflammatory response in AD. The reduction in pro-inflammatory cytokine secretion by human astrocytes using the selective CK2 inhibitor CX-4945 indicates that CK2 could be a potential target to modulate neuroinflammation in AD.

**Electronic supplementary material:**

The online version of this article (doi:10.1186/s12974-015-0470-x) contains supplementary material, which is available to authorized users.

## Background

Alzheimer’s disease (AD) is currently the most common neurodegenerative disease and is characterized by memory loss and cognitive impairment. Pathological hallmarks of AD include extracellular deposits of amyloid beta (Aβ), as well as intracellular accumulations of hyperphosphorylated tau in neurofibrillary tangles (NFTs) and neuropil threads [1]. In approximately 80 % of AD cases, Aβ deposits are also observed in parenchymal and leptomeningeal vessels, which is referred to as cerebral amyloid angiopathy (CAA) [2]. Two types of CAA can be distinguished: Aβ accumulation in the walls of both larger vessels and capillaries (CAA type I or capCAA) and Aβ accumulation only present in the walls of larger vessels (CAA type II) [3]. It has been reported that capCAA is associated with a neuroinflammatory response resulting in alterations of the blood-brain barrier (BBB) which contribute to AD pathology [2].

In AD, neuroinflammation is associated with an increase of activated complement proteins and cytokines, such as interleukin 6 (IL-6) and monocyte chemotactic protein-1 (MCP-1; also referred to as chemokine (C-C motif) ligand 2 (CCL2)), and activated microglia and astrocytes [4, 5]. Interestingly, these markers are already present in the cerebral cortex at early stages of AD pathology [6–8]. Among the genes that increase the risk of developing AD, several are associated with inflammation, for instance TREM2, CD33, CR1 and CLU [9–14]. As such inflammation is considered an integral part of AD pathology [9, 10] and is characterized as a local activation of the innate immune response, referred to as neuroinflammation. It is thought that the neuroinflammatory response in AD is aimed at eliminating injurious stimuli and restoring tissue integrity.

For the development of therapeutic strategies, it is essential to elucidate the underlying processes and signalling pathways that drive AD pathology [15]. Protein kinases are major targets for investigation as they are involved in most cellular processes and are key regulators of multiple signalling pathways [16]. In addition, many protein kinases are drug-able and as such potential therapeutic targets for the treatment of AD [17].

One of the first protein kinases identified in an AD brain was protein kinase CK2, formerly known as casein kinase II [18, 19]. CK2 consists of two catalytic α subunits (39–45 kDa) and two regulatory β subunits (26 kDa) and is constitutively active [20]. There are at least three different isoforms for the catalytic α subunit: α, α’ and α” [21, 22]. CK2 utilizes both adenosine triphosphate (ATP) and guanosine triphosphate (GTP) as phosphate donors [19, 23, 24] and has a broad spectrum of over 300 protein substrates [23]. In addition to phosphorylating serine and threonine residues, CK2 is capable to phosphorylate tyrosine residues, thereby making it a dual-specificity kinase [24, 25].

The mechanisms of CK2 regulation are poorly understood. Emerging evidence suggests a potential role for CK2 during pathogeneses associated with inflammation [26]. CK2 regulates the activity of several key transcription factors implicated in inflammation, e.g. nuclear factor kappa-light-chain-enhancer in activated B cells (NF-κB) [27]. In turn, an increasing number of studies report the regulation of CK2 activity by cytokines and other pro-inflammatory agents. Lipopolysaccharide (LPS) for example, a major inducer of pro-inflammatory cytokine expression, has been found to induce CK2 activity in murine RAW264.7 macrophages [28]. Tumour necrosis factor-α (TNF-α) has been shown to stimulate CK2 activity in Swiss L929, 3T3 and human cervical carcinoma HeLa cells [26, 29, 30], and interleukin-1 (IL-1) has been reported to activate CK2 in intestinal epithelial cells [31]. The transforming growth factor-β (TGF-β) has been shown to stimulate CK2 activity in murine mesangial cells and in macrophages [32, 33]. Furthermore, interferon-γ (IFN-γ) induced CK2 activity in macrophages [34, 35]. Understanding the regulation of CK2 signalling and its role in inflammatory pathways will be essential for the design of novel therapeutic strategies for diseases associated with inflammation such as AD.

A number of chemical compounds have been analysed as inhibitors for CK2 [36, 37], like the ATP-competitive 4,5,6,7-tetrabromo-1*H*-benzotriazole (TBB) [38], which has been used widely as a molecular probe to elucidate the functional role of CK2. Another promising CK2 inhibitor is the small molecule CX-4945, also known as Silmitasertib [39]. CX-4945 is a highly selective ATP-competitive inhibitor of both CK2α and CK2α’ catalytic subunits [39, 40]. It is orally administered and is currently the only CK2 inhibitor that is being evaluated in clinical trials for the treatment of many cancer types [40]. In phase I clinical trials for patients with different solid tumours, adverse effects of CX-4945 were generally mild to moderate, demonstrating that CX-4945 can be safely administered to humans [41].

In this study, we have investigated the role of CK2 in the hippocampus and temporal cortex of AD patients and non-demented controls. In addition, we investigated the effect of two CK2 inhibitors on IL-6 and MCP-1 secretion in stimulated human primary astrocytes and U373 cells. Our study provides support for a role of CK2 in neuroinflammation suggesting that CK2 could act as a potential target for modulating the inflammatory response in AD.

## Methods

### Case selection

Brain samples were obtained from The Netherlands Brain Bank (NBB), Netherlands Institute for Neuroscience (Amsterdam, The Netherlands). All donors or their next of kin gave written informed consent for a brain autopsy and the use of the material and clinical information for research purposes according to the Declaration of Helsinki. This work was approved by the ethics committee of the NBB. Dementia status at death was determined on the basis of clinical information available during the last year of life and neuropathological diagnosis using (immuno)histochemical stainings (haematoxylin and eosin, Bodian and/or Gallyas silver stainings, methenamine silver staining and immunohistochemial stainings for Aβ, tau, α-synuclein, TDP and P62. Analysis of formalin-fixed and paraffin-embedded tissue from different parts of the brain was performed, including the frontal cortex (F2), temporal pole cortex, parietal cortex (superior and inferior lobule), occipital pole cortex, amygdala and the hippocampus, essentially CA1 and entorhinal area of the parahippocampal gyrus. Staging of AD pathology was evaluated according to a modified assessment of Braak and Alafuzoff [42] and the Aβ staging of Thal [43, 44]. Severity of dementia was determined using the Global Deterioration Scale of Reisberg (GDS) [45]. Cases with and without clinical neurological disease diagnosis were processed identically. Of all cases used for this study, age, sex, clinical diagnosis, stage of AD pathology (Braak stage and phase of Aβ deposition), CERAD score [46] and post mortem delay (PMD) are listed in Table 1. We compared non-demented cases with low AD pathology, including seven cases of primary age-related tauopathy (PART [47, 48]; Table 1: 4, 15, 17, 26, 27, 30, 31) and cases with symptomatic, late-stage AD.

**Table 1:** List of AD and CON cases used for this study

Cases used for Western blotting							
A. Hippocampus							
Case number	Braak stage (NFT)	Phase of Aβ deposits	CERAD neuritic plaque score	Sex	Age	PMD	Pathological diagnosis
1	0	O	0	M	51	7.45	CON
2	1	B	0	M	85	7.05	CON
3	1	A	0	F	60	7.30	CON
4	2	O	0	F	81	5.30	CON
5	3	A	0	F	91	5.20	CON
6	4	C	2	F	91	6.05	AD
7	5	C	2	F	89	4.40	AD
8	5	C	2	M	81	NA	AD
9	5	B	2	F	69	7.10	AD
10	6	C	3	F	69	5.30	AD
11	6	C	3	F	68	3.50	AD
12	6	C	3	F	67	5.50	AD
B. Temporal cortex							
13	0	B	0	M	74	7.45	CON
14	0	O	0	M	66	7.45	CON
15	1	O	0	M	77	8.25	CON
16	1	B	0	M	71	5.45	CON
17	2	O	0	F	93	5.50	CON
18	2	A	0	F	85	4.40	CON
19	3	C	1	F	82	6.05	AD
20	4	C	2	M	64	6.00	AD
21	5	C	3	F	71	4.15	AD
22	6	C	3	M	65	8.50	AD
23	6	C	3	F	64	3.40	AD
24	6	C	3	M	69	5.30	AD
C. Cases used for immunohistochemistry							
Case number	Braak stage (NFT)	Phase of Aβ deposits	CERAD neuritic plaque score	Sex	Age	PMD (hrs)	Pathological diagnosis
*25	0	O	0	M	74	8.05	CON
26	1	O	0	F	75	5.25	CON
27	1	O	0	M	82	5.50	CON
28	1	B	0	F	85	7.05	CON
29	1	B	0	F	73	7.45	CON
30	1	O	0	F	60	6.50	CON
31	1	O	0	M	78	17.40	CON
32	2	B	0	F	84	6.05	CON
33	2	B	0	F	83	4.40	CON
34	5	C	2	F	84	4.50	AD
35	6	C	3	F	62	4.45	AD
*36	6	C	3	M	60	6.15	AD
37	6	C	3	F	82	5.30	AD
38	6	C	3	M	74	7.40	AD
39	6	C	3	F	81	6.00	AD
40	6	C	3	M	73	6.15	AD
41	6	C	3	M	74	5.35	AD
*42	6	C	3	F	82	6.00	AD
*43	6	C	3	F	67	6.05	AD
*44	5/6	NA	NA	M	76	24.00	AD, CAA type 2
*45	5	C	3	M	65	7.20	AD, CAA type 2
46	6	C	3	F	87	8.00	AD, CAA type 2

*CON* control case, *AD* Alzheimer’s disease case, *NFT* neurofibrillary tangles, *CERAD* consortium to establish a registry for Alzheimer’s disease, *PMD* post mortem delay, *hrs* hours, *CAA* cerebral amyloid aniopathy, *NA* not available

**Fig. 1:**
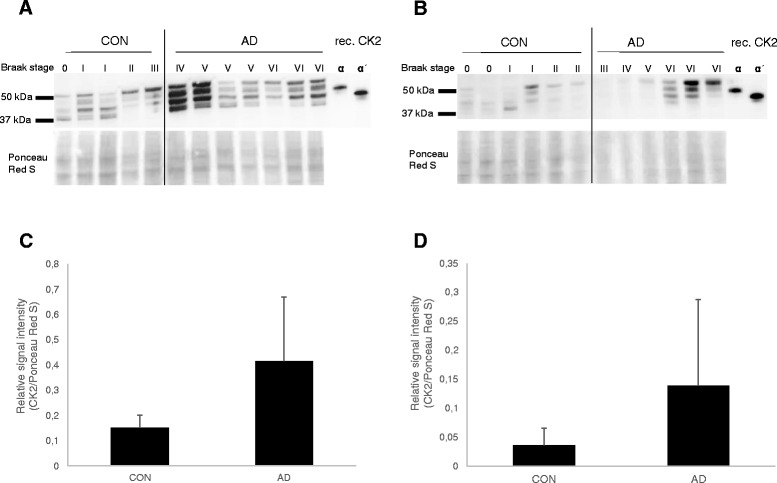
Expression of CK2 in the hippocampus and temporal cortex. Protein expression of CK2 was assessed by Western blot analysis using mouse anti-CK2 which detects both CK2α and CK2α’ (see Additional file 2: Figure S1). **a** Western blot analysis of brain extracts from the hippocampus. **b** Western blot analysis of brain extracts from the temporal cortex. AD and non-demented control (CON) cases analysed by Western blotting are listed in Table 1. Braak stages are indicated, and recombinant CK2α and CK2α’ were used as positive controls. Ponceau red staining was used in order to determine the relative protein load on the blotting membrane. **c** Quantification of CK2α/α’ expression in the hippocampus of AD and control cases. **d** Quantification of CK2α/α’ protein expression in the temporal cortex. Shown are mean levels +/− SD

All cases were used for quantification (Figs. 1 and 3) except when indicated with *

### Preparation of brain tissue lysates

Twenty 10-μm-thick frozen tissue slices of the hippocampus and temporal cortex were cut at −20 °C. Brain extracts were prepared by adding 100 mg wet-weight brain tissue to 1 ml cold M-PER (Mammalian Protein Extraction Reagent, Thermo Scientific, MA, USA) lysis buffer containing Protease Inhibitor Cocktail (Roche, Basel, Switzerland) and Phosphatase Inhibitor Cocktail (Roche). Samples were left on ice for 30 min and after centrifugation (10 min, 4 °C, 10 000×*g*), the supernatant was collected, snap frozen in 100 μl aliquots and stored at −80 °C until further analysis. The protein concentration was determined using the Bradford Lowry Assay (Bio-Rad Protein Assay, Hercules, CA, USA) with bovine serum albumin (BSA, Roche) as standard.

### Western blotting

Sample buffer (Thermo Scientific) was added to the protein lysates and heated for 5 min at 95 °C. Proteins were separated by SDS-PAGE using a polyacrylamide gradient gel in running buffer (25 mM Tris, 192 mM glycine, 0.1 % SDS, pH 8.3, Bio-Rad). In a separate experiment, whole cell lysates of cultured primary astrocytes and U373 cells were lysed with sample buffer in a 1:1 ratio and heated for 5 min at 95 °C. Proteins were separated on a custom cast acrylamide gel (10 %) and electrophoretically transferred onto a nitrocellulose membrane (0.2 μm; Whatman, Protran™, Thermo Scientific) using transfer buffer (25 mM Tris, 192 mM glycine, 20 % methanol, Bio-Rad). Ponceau red S solution was used as a loading control. Membranes were blocked for 1 h in Tris-buffered saline (50 mM Tris pH 7.5, 0.15 M NaCl, 0.1 % Tween-20, pH 8.3) containing 5 % BSA (Roche) and incubated over night with primary anti-CK2α antibody (1:500, mouse monoclonal, SC-12738, Santa Cruz Biotechnology, CA). Subsequently, blots were incubated with a secondary antibody linked to horseradish peroxidase (HRP-anti-rabbit IgG or HRP-anti-mouse IgG, 1:1000, Dako, Glostrup, Denmark) overnight at room temperature. Immunoreactive bands were detected with an enhanced chemiluminescence reagent (ECL Plus, GE Healthcare, Buckinghamshire, UK). The intensity of the bands was quantified using MacBiophotonics ImageJ (version 1.48k). Data was expressed as relative signal intensities (CK2α/Ponceau red S) of the individual samples. An overview of the antibodies used in this study is given in Table 2. Recombinant CK2α and CK2α’ (100 ng; Millipore, Dundee, UK) were used to determine the selectivity of the antibodies.

**Table 2:** Overview of antibodies

Antibodies used for Western blotting			
Antibody (antigen)	Species	Dilution	Source
CK2α	Mouse	1:500	Santa Cruz Biotechnology, CA, USA
CK2α'	Goat	1:500	Santa Cruz Biotechnology, CA, USA
HRP anti-mouse	Rabbit	1:1000	Dako, Glostrup, Denmark
HRP anti-goat	Rabbit	1:1000	Dako, Glostrup, Denmark
Antibodies used for immunohistochemistry			
Tau pSer202, pThr205 (AT8)	Mouse	1:800	Pierce, Rockford, IL, USA
Aβ (IC-16)	Mouse	1:200	Prof. C. Korth, Düsseldorf, Germany
GFAP	Mouse	1:50	Monosan, Uden, The Netherlands
CK2α	Mouse	1:50	Santa Cruz Biotechnology, CA, USA

### Immunohistochemistry

For immunohistochemical analysis, frozen 5-μm-thick sections were mounted on coated glass slides (Menzel Gläser Superfrost PLUS, Thermo Scientific). Sections were fixed by immersion in acetone for 10 min, followed by washing in phosphate-buffered saline (PBS, pH 7.4). Between the subsequent incubation steps, sections were washed extensively with PBS. Incubation with the primary antibodies was overnight at 4 °C. Mouse monoclonal anti-GFAP (1:50, Monosan, Uden, The Netherlands), mouse monoclonal anti-CK2α (1:50, Santa Cruz Biotechnology, CA), mouse monoclonal anti-phospho-tau [49] (AT8 for Tau pSer202 and pThr205, 1:800, Pierce Biotechnology) and mouse monoclonal anti-Aβ (IC16 1:200, Prof. C. Korth, Heinrich Heine University Düsseldorf, Germany) were used. In addition, the mouse monoclonal anti-CK2α antibody was tested on formalin-fixed, paraffin-embedded tissue (Additional file 1: Figure S3). The antibodies (Table 2) were diluted in antibody diluent (Immunologic, Duiven, The Netherlands). Omission of the primary antibodies served as a negative control. Secondary EnVison™ HRP goat anti-rabbit/mouse antibody (EV-GαM^HRP^, Dako) incubation was for 30 min at room temperature. The secondary antibody was detected using 3,3-diaminobenzidine (Dako). Sections were counterstained with haematoxylin for 1 min to visualize the nuclei of the cells, dehydrated and mounted using Quick-D mounting medium (BDH Laboratories Supplies, Poole, England). For Congo red staining, sections were incubated with 50 ml saturated NaCl solution (0.5 M NaCl in 80 % ethanol, supplemented with 0.5 ml 1 % NaOH) after dehydration. Sections were transferred to saturated 50 ml 0.5 % Congo red solution (VWR International, Leuven, Belgium) for 20 min and mounted with Quick-D mounting medium. CK2 immunoreactivity was determined blinded to the pathological and clinical diagnosis. Full images of six representative microscopic fields were obtained using a Zeiss light microscope equipped with a digital camera, a ×12 ocular and a ×10 objective. The percentage of the area showing immunoreactivity for a specific antibody (area fraction) was determined using MacBiophotonics Image-J software (version 1.48). Student’s *t* test was used to determine differences between AD and CON cases. Results are expressed as mean ± standard deviation (SD). A *p* value of <.05 was considered significant.

### Localisation with triple immunofluorescence

To investigate co-localization of CK2, amyloid and astrocytes, frozen brain tissue sections were dried and submerged in 100 % acetone for 10 min at room temperature and subsequently incubated with thioflavin S solution (100 mg/ml, Sigma, St. Louis, USA) for 5 min to stain amyloid fibrils. The sections were washed with 100 % ethanol and PBS, followed by incubation with Normal Goat Serum (NGS, 1:10 dilution, Dako) for 10 min to block a specific binding of the antibodies. Then, the sections were incubated with a mixture of primary antibodies: CK2α (1:50, Santa Cruz Biotechnology) and GFAP (1:300, Monosan) overnight at 4 °C. Subsequently, sections were washed with PBS and incubated with a mixture of secondary antibodies: EV-GαM^HRP^ (Dako) and GαR-Cy5 (1:100, Jackson ImmunoResearch Laboratories, West Grove, PA) for 1 h. The sections were washed with PBS and developed with rhodamine/tyramide intensification (1:3000, 0.01 % H_2_O_2_) for 5 min. To block autofluorescence, the sections were incubated with Sudan Black (0.3 %, diluted in 70 % ethanol). Sections were mounted in plain 80 % Tris-buffered glycerol.

### In vitro functional assays

Adult primary human astrocytes were isolated from brain specimens obtained at autopsy through The Netherlands Brain Bank and cultured as described previously [50, 51]. Primary astrocyte cultures from clinically diagnosed AD patients and control cases (patients with epilepsy) were included in this study. No differences in functionality were observed between the astrocytes from different cases. All experiments were performed at least in triplicates. The human glioblastoma cell line U373 (HTB-17) was obtained from American Type Culture Collection (ATCC, Rockville, MD, USA). Cells were grown at 37 °C as a monolayer in culture medium (Dulbecco’s modified Eagle’s medium (DMEM) and Ham’s F10 Nutrient Mixture (HAM-F10) 1:1, supplemented with 2 mM l-glutamin (Gibco, Waltham, MA, USA), 10 % (*v*/*v*) foetal calf serum (FCS, Integro, Zaandam, The Netherlands), 100 U/ml penicillin (Gibco) and 50 μg/ml streptomycin (Gibco) with 5 % CO_2_ in culture flasks (Greiner, Alphen a/d Rijn, The Netherlands)).

For stimulation/inhibition experiments, U373 cells and primary astrocytes were trypsinized (Sigma) and transferred to 24-well plate (Nunc, Roskilde, Denmark) at 5 × 10^4^ cells/well in culture medium. Stock solutions of the inhibitors tetrabromobenzotriazole (64 mM, TBB, Sigma) and 5-(3-chlorophenylamino)benzo[c][2,6]-naphthyridine-8-carboxylic acid (CX-4945, 100 mM, Medchem, Leiden, The Netherlands) were prepared in dimethylsulfoxide (DMSO, Merck, Kenilworth, NJ, USA). The inhibitor was added to the cultures 1 h prior to co-incubation with either human recombinant IL-1β (10 U/ml, Peprotech, London, UK) or TNF-α (100 ng/ml, Immuno Tools, Friesoythe, Germany) for 24 h in culture medium containing 10 % foetal bovine serum (FCS). IL-1β/TNF-α was added directly onto the culture medium which contained the inhibitor. The final DMSO concentration never exceeded 0.03 % for TBB and 0.01 % for CX-4945. Cell culture medium was collected by centrifugation and stored at −20 °C until further analysis. The monocyte chemoattractant protein-1 (MCP-1) was determined using the DuoSet MCP-1 enzyme-linked immune sorbent assay (ELISA) (R&D Systems Europe, Abingdon, UK), while for interleukin-6 (IL-6), the Pelipair IL-6 ELISA kit (Sanquin, Amsterdam, The Netherlands) was employed. The effect of TBB and CX-4945 inhibitor on cell viability was determined using the 3-(4,5-dimethylthiazol-2-yl)-2,5-diphenyltetrazolium bromide (MTT/Formazan) assay [52]. For statistical analysis, unpaired *t* tests were performed.

### Fluorescent immunocytochemistry of cultured cells

Primary astrocytes and U373 cells were stimulated as described in 2.6 and cultured on a borosilicate glass slide (VWR International, Amsterdam, The Netherlands) in a 24-well plate. After 24 h, culture medium was collected and the glass slides with the cells were washed with PBS. After fixation in 4 % formaldehyde (Klinipath, Duiven, The Netherlands) for 15 min, cells were washed with PBS 0.1 % Triton (Merck) for 30 min. Cells were incubated with the CK2α antibody (Santa Cruz, 1:50 dilution in PBS 0.05 % Triton/0.5 % BSA) overnight at room temperature while shaking. After washing for three times with 500 μl PBS/0.1 % Triton, the cells were incubated with the secondary fluorescently labelled antibody (Alexa fluor 594, Invitrogen) in a dilution of 1:1000 for 90 min in a dark environment on a shaker. After washing with PBS, cell nuclei were stained with DAPI (1:10.000 dilution in PBS, Life Technologies, Amsterdam, The Netherlands) for 10 min. The cells were washed with PBS and transferred to a microscopy slide (Menzel, superfrost colour, Thermo Scientific) using an 80 % Tris-HCl buffered glycerol solution (pH 7.5).

## Results

### CK2 protein levels are increased in AD brain

CK2 protein levels were assessed by Western blot analysis using brain tissue extracts of the temporal cortex and hippocampus of AD and non-demented control cases (CON; Table 1). Recombinant human CK2α and CK2α’ proteins were used to determine the specificity of the antibodies. Mouse monoclonal anti-CK2α detected the α-isoform as well as the α’-isoform (indicated by CK2α/α’ throughout the manuscript), whereas the goat polyclonal anti-CK2α’ only detected the α’-isoform (Additional file 2: Figure S1). In protein lysates obtained from post mortem human hippocampus (Fig. 1a) and temporal cortex (Fig. 1b), four bands between 37 and 50 kDa were detected using the CK2α/α’ antibody. Based on bands obtained with the recombinant proteins, the second and third band observed with the brain extracts were assigned to CK2α and CK2α’, respectively. It is likely that the other two bands correspond to different isoforms of CK2 [21]. Increased levels of CK2α/α’ compared to controls were observed in the hippocampus (Fig. 1c) and temporal cortex (Fig. 1d) of AD brains. In the hippocampus, increased levels of CK2 became apparent at Braak stage 4 (Fig. 1a), while in the temporal cortex, a prominent increase was observed in Braak stage 6 (Fig. 1b). The full Western blots are shown in Additional file 3: Figure S2.

### CK2α/α’ immunoreactivity is increased in AD patients and is associated with astrocytes, amyloid fibrils and blood vessels

**Fig. 2:**
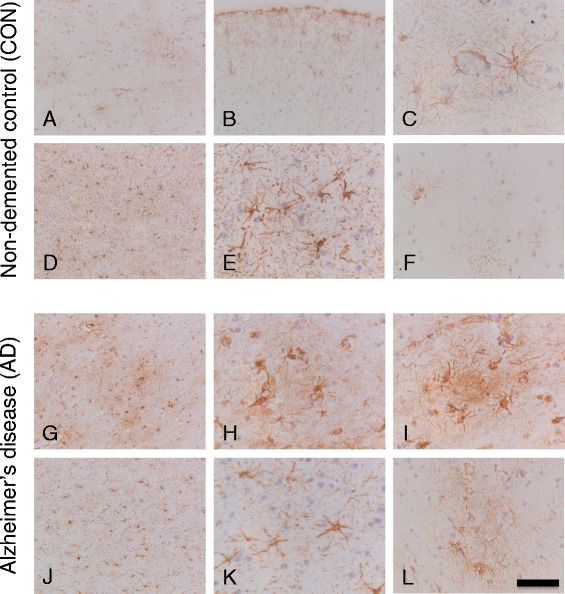
Immunohistochemical detection of CK2α/α’ in control and AD brain. Shown are representative immunohistochemical stainings for CK2α/α’ of the mid-temporal cortex and hippocampus of a control (**a**–**f**, Braak stage 0, Table 1C case #25) and two AD cases (Braak stage VI, Table 1C cases **g**–**i** #35 and **j**–**l** #36). **a** CK2α/α’ immunoreactivity in the grey matter of the mid-temporal cortex in a control case. **b** CK2α/α’ immunoreactivity in the subpial layer of the mid-temporal cortex in a control cases. **c** Detailed picture of CK2α/α’ immunoreactivity in astrocytes around a blood vessel in the grey matter of the mid-temporal cortex in a control case. **d** Overview of CK2α/α’ immunoreactivity in astrocytes in the white matter (temporal cortex) of a control case. **e** Detailed picture of CK2α/α’ immunoreactivity in the white matter of a control case. **f** CK2α/α’ immunoreactivity in the CA1 region of the hippocampus of a control case. **g** CK2α/α’ immunoreactivity in the grey matter of the mid-temporal cortex in an AD case. **h**, **i** Detailed pictures of clusters of astrocytes with CK2α/α’ immunoreactivity in the grey matter of the mid-temporal cortex in an AD case. **j** Overview of CK2α/α’ immunoreactivity in the white matter (temporal cortex) of an AD case. **k** Detailed picture of CK2α/α’ immunoreactivity in astrocytes in the white matter of an AD case. **l** CK2α/α’ immunoreactivity in the CA1 region of the hippocampus of an AD case. Immunohistochemical detection was performed using DAB (*brown*) and nuclei were stained with haematoxylin (*blue*). *Scale bars* **a**, **b**, **d**, **g** and **j** 200 μm; **f** and **l** 100 μm; **c**, **e**, **h**, **i** and **k** 50 μm

**Fig. 3:**
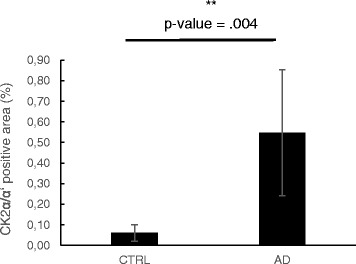
Quantification of CK2α/α’ immunoreactivity in the temporal cortex of CON and AD patients. Quantification of CK2α/α’ positivity in the temporal cortex (grey matter) of AD patients (*n* = 8) and non-demented controls (CON, *n* = 8). Mean levels (± SD) of the area density are expressed as percentage of immunoreactive area of the total area. *p value <.05

The localization and distribution of CK2 in the temporal cortex and hippocampus of AD and control cases was assessed using immunohistochemistry on fresh-frozen tissue. Representative images are shown in Fig. 2. Similar results were obtained with CK2 immunohistochemistry on formalin-fixed paraffin-embedded brain tissue (Additional file 1: Figure S3). CK2α/α’ staining showed a star-like shape and was found in both control and AD cases. Based on the morphological appearance, immunoreactive cells could be identified as astrocytes. In control cases, CK2α/α’ immunoreactivity was specifically observed in the grey matter of the mid-temporal cortex (Fig. 2a and b) around blood vessels (Fig. 2c) and in the white matter (Fig. 2d and e) and was less prominent in the hippocampal CA1 area (Fig. 2f). There was no difference between the CK2α/α’ levels in the white matter of AD cases (Fig. 2j, k) and controls. In contrast, CK2α/α’ immunoreactivity was increased in AD, prominently in the grey matter regions of the temporal cortex (Fig. 2g). The CK2α/α’ immunoreactivity in the temporal cortex of 16 patients (8 control and 8 AD cases; Table 1) was determined and quantified. A significant increase of CK2α/α’ in AD brains compared to controls (*p* value <.05; Fig. 3) was observed. An increase of CK2α/α’ in AD compared to control was also detected in the CA1 region of the hippocampus (Fig. 2f, l). Interestingly, CK2α/α’ immunoreactivity in the cortical areas showed a clustered distribution (Fig. 2h, i), which might suggest the presence of amyloid depositions.

**Fig. 4:**
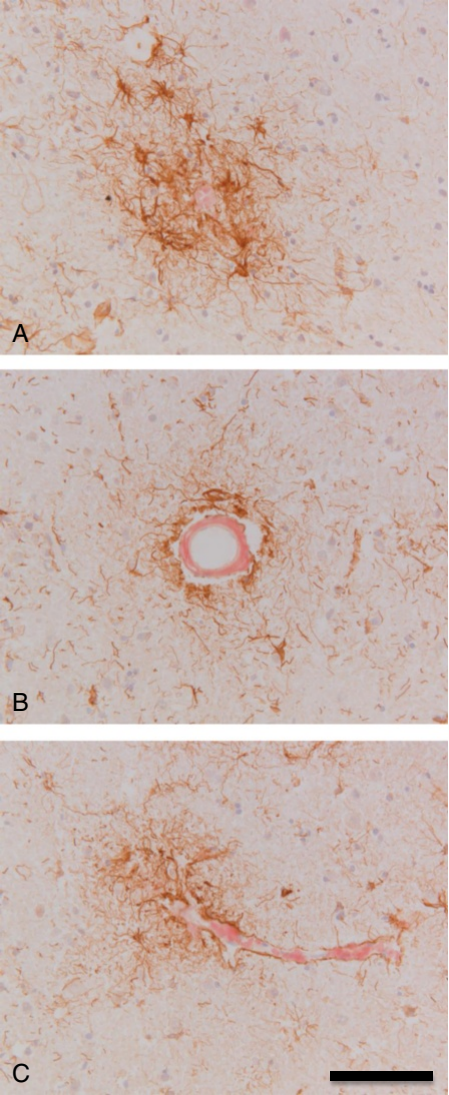
Association of CK2α/α’ with amyloid deposits in AD. Representative pictures are shown of combined Congo red and immunohistochemical stainings for CK2α/α’ of the cortex of two AD cases (**a**: Braak stage V, Table 1C case #45; **b** + **c**: Braak stage V/VI, Table 1C case #44). **a** Association of astrocytes immunoreactive for CK2α/α’ with a Congo red positive amyloid plaque. **b** Astrocytes immunoreactive for CK2α/α’ associated with a Congo red positive blood vessel. **c** Association of CK2α/α’ immunoreactivity with a Congo red positive capillary. Immunohistochemical detection was performed using DAB (*brown*) and nuclei were stained with haematoxylin (*blue*). Scale bar A-C 100 μm

**Fig. 5:**
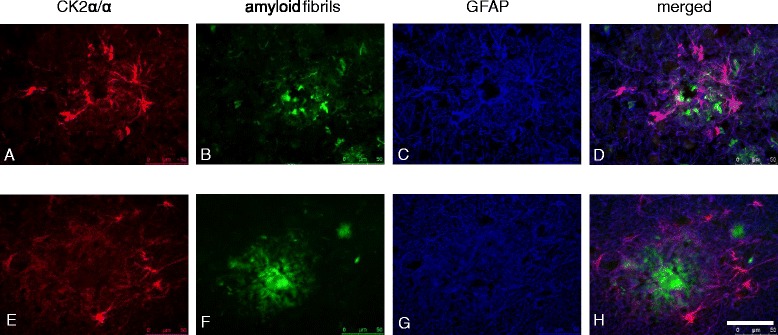
Triple fluorescent stainings of CK2α/α’, amyloid fibrils and astrocytes in temporal cortex and hippocampus of AD patients. Shown are representative immunohistochemical stainings for CK2α/α’ of the mid-temporal cortex (**a**–**d**, Braak stage VI, Table 1C case #43) and hippocampus (**e**–**h**, Braak stage VI, Table 1C case #42) of two AD patients. **a**, **e** CK2α/α’ immunoreactivity, **b**, **f** amyloid fibrils (Thioflavin) and **c**, **g** astrocytes (GFAP) in the temporal cortex (**a**–**d**, Braak stage VI, Table 1C case #43) and hippocampus (**e**–**h**, Braak stage VI, Table 1C case #42) of AD patients. Immunofluorescent detection was performed using CKα/α’ (*red*), amyloid fibrils (*green*) and astrocytes (GFAP) (*blue*). Scale bars A–H 50 μm

To confirm the increased appearance of CK2α/α’ immunoreactivity around amyloid deposits, we performed co-stainings of CK2α/α’ with different amyloid dyes. Increased CK2α/α’ immunoreactivity was observed around Congo red positive amyloid plaques (Fig. 4a). In addition, CK2α/α’ immunoreactivity was also associated with different types of cerebral amyloid angiopathy, with large blood vessels containing amyloid (Fig. 4b) as well as amyloid containing capillaries (Fig. 4c). CK2α/α’ immunoreactivity was also observed around Thioflavin S positive amyloid plaques in AD temporal cortex and hippocampus (Fig. 5). To confirm that CK2α/α’ immunoreactive cells were indeed astrocytes, co-labelling was performed with the astrocytic marker GFAP. This indicates that CK2α/α’ co-localizes with astrocytes present around amyloid deposits in the hippocampus and temporal cortex of AD patients (Fig. 5).

### CK2 activity in human primary astrocytes and U373 cells is inhibited by CX-4945

**Fig. 6:**
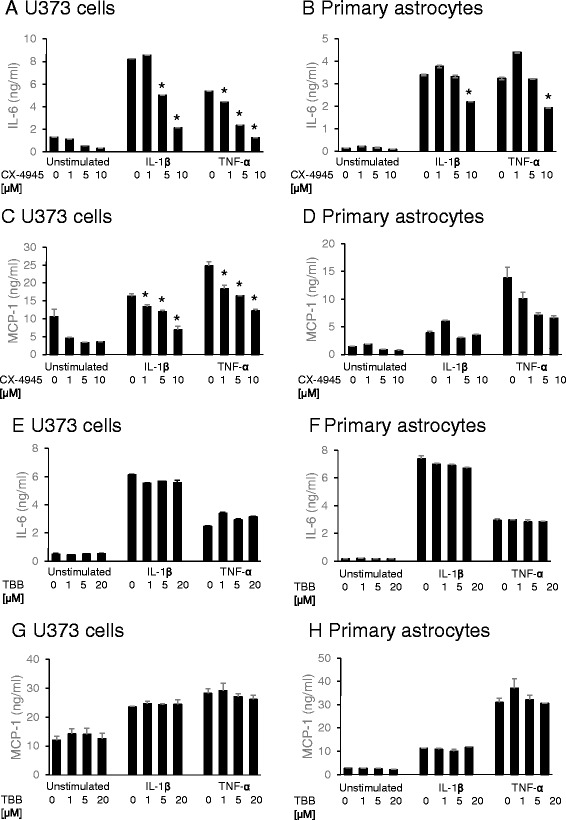
Effect of CK2 inhibitors on IL-6 and MCP-1 secretion. U373 cells and human primary astrocytes were stimulated with either IL-1β (10 U/ml) or TNF-α (100 ng/ml) for 24 h in the absence or presence of different concentrations of CX-4945 (**a**) or TBB (**b**). IL-6 and MCP-1 levels in the culture supernatants were determined by ELISA. The stars indicate significant difference (*p* value <.05) between the condition without inhibitor, labelled with 0 μM, and the corresponding condition with either 1, 5 or 10 μM CX-4945 (**a**–**d**) or 1, 5, 20 μM TBB (**e**–**h**)

Since CK2 primarily co-localizes with astrocytes (Fig. 5), CK2 activity was assessed in adult human primary astrocytes and in the astrocytoma cell line U373 to investigate the role of CK2 in neuroinflammation. Two ATP-competitive inhibitors were chosen for functional analysis of CK2 activity, namely TBB, one of the most studied CK2 inhibitors [53] and the highly selective CK2 inhibitor CX-4945. The MTT assay showed that the mitochondrial activity, which was used as a marker for cell viability, was not affected in the presence of either TBB or CX-4945 in concentrations up to 20 and 10 μM, respectively (data not shown). Since we observed prominent presence of CK2 in reactive astrocytes associated with amyloid deposits, we chose to use an inflammatory model resembling the situation around the amyloid deposits [54, 55]. U373 cells and human astrocytes were stimulated with IL-1β or TNF-α, which induced the secretion of MCP-1 and IL-6 (Fig. 6).

The stimulation of U373 cells with IL-1β (24 h) in the presence of CX-4945 resulted in a significant decrease of the levels of IL-6 in the culture supernatant, i.e. to 50 % at 5 μM and 25 % at 10 μM of inhibitor concentration (Fig. 6a). The TNF-α stimulated U373 cells (24 h) were slightly more responsive to inhibition compared to IL-1β stimulated cells. The amount of IL-6 in the medium was reduced to 40 % at 5 μM (Fig. 6a). The amount of secreted MCP-1 by U373 cells was decreased in the presence of CX-4945 to 80 % at a concentration of 1 μM, both for IL-1β and TNF-α stimulated cells (Fig. 6c).

Similar results were obtained with human primary astrocytes. We observed a significant decrease of IL-6 in the culture supernatant of stimulated astrocytes in the presence of 10 μM CX-4945, i.e. to 45 % for IL-1β-stimulated astrocytes and for TNF-α-stimulated astrocytes, a decrease to 50 % (Fig. 6b). The MCP-1 secretion was effected more upon treatment with CX-4945 compared to IL-6 secretion in the culture supernatant. For IL-1β stimulated astrocytes, the amount of MCP-1 was reduced to 40 and 25 % in the presence of 5 and 10 μM CX-4945, respectively (Fig. 6d). For TNF-α stimulated astrocytes, a decrease to 50 and 40 % of MCP-1 was observed in the presence of 5 and 10 μM CX-4945, respectively. No significant reduction of IL-6 and MCP-1 levels in the culture supernatant of stimulated U373 cells of human primary astrocytes was observed after incubation with TBB (Fig. 6e-h).

**Fig. 7:**
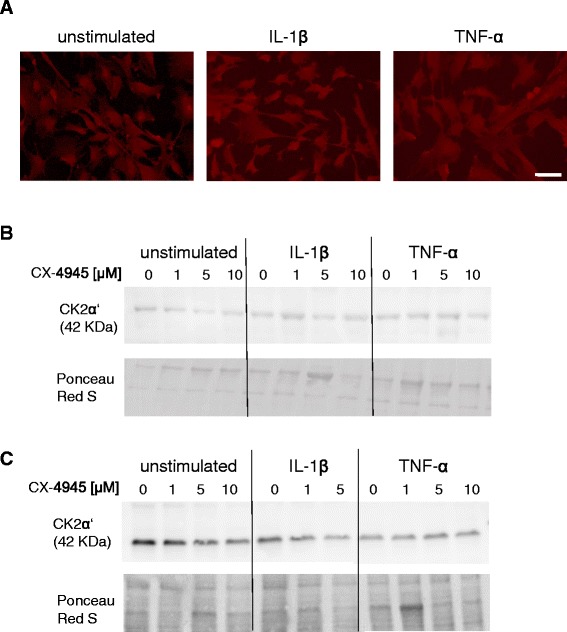
CK2α’ expression levels in primary astrocytes after stimulation with IL-1β or TNF-α in the presence of CX-4945. **a** Astrocytes were immunostained for CK2 (goat anti-CK2α’ antibody) in *red* (Alexa 594) without stimulation and after stimulation with IL-1β or TNF-α. Scale bar for all panels 50 μm. **b** CK2α’ expression levels were determined by Western blotting (goat anti-CK2α’ antibody). Human primary astrocytes or **c** U373 cells were cultured without stimulus, with IL-1β or TNF- α for 24 h in the absence or presence of CX-4945 (1–10 μM)

In order to investigate if the observed changes occurred in the presence of CX-4945 are changes in the activity or the protein level of CK2α/α’, Western blotting analysis was performed with primary human astrocytes and U373 cells. Using the monoclonal mouse antibody detecting both CK2α and CK2α’, we were not able to detect CK2 by Western blotting or immunofluorescence of cultured cells. No changes in protein levels of CK2α’ were observed upon stimulation of primary astrocytes (Fig. 7a) or U373 cells (Fig. 7b) stimulated with IL-1β or TNF-α, nor in the presence of CX-4945. Furthermore, the cellular localization of CK2α’ in both cytoplasm and the cell nuclei [56, 57] as well as protein levels of CK2α’ in primary astrocytes remained unchanged after stimulation with either IL-1β or TNF-α (Fig. 7c). The same observations were made with the U373 cells (data not shown). We conclude that CK2 activity rather than expression is involved in modulating the IL-1β or TNF-α induced MCP-1 and IL-6 secretion.

## Discussion

In this study, we report that the amount of CK2α/α’ is increased in hippocampus and temporal cortex of AD patients. CK2α/α’ co-localizes with astrocytes, and, in AD cases, CK2α/α’ immunoreactive astrocytes surround amyloid deposits. The selective CK2 inhibitor CX-4945 reduces the IL-1β or TNF-α induced secretion of MCP-1 and IL-6 both in human primary astrocytes and U373 cells in a dose-dependent manner without affecting the protein expression levels of CK2α’.

CK2 has been suggested to potentially play different roles in AD, during synaptic plasticity [58, 59], APP processing [60–62], tau accumulation [63, 64] and insulin signalling [65]. There are contradictory reports on the expression levels of CK2 observed in AD brain tissue. Decreased CK2 activity has been reported in the frontal cortex of AD patients [18, 66, 67]. In contrast, increased CK2 expression, preceding tau accumulation and tangle formation, has been observed during human AD pathogenesis [68]. Initial studies reported that CK2 is expressed in neurons [56, 57]. More recently, Kramerov et al. showed that CK2 is expressed in astroglial cells of normal and neovascularized retina, thereby suggesting that CK2 might be useful as a new immunohistochemical marker for astrocytes [69, 70].

To study the involvement of CK2 in AD pathology, we compared symptomatic, late-stage AD cases (Braak V + VI) with non-demented and pre-clinical AD cases (Braak 0–II), including cases of PART. CK2 levels showed a prominent increase with Braak stage. We were able to confirm the results of Kramerov et al. and show that CK2 is present in astrocytes in AD and control temporal cortex and hippocampal brain tissue. Previous reports have shown that astrocytes are involved at early stages of AD pathology, for example, the uptake and removal of Aβ [71, 72] and that the inflammatory response is a driving factor in AD pathology. Our data suggests a role for CK2 in astrocyte function during the neuroinflammatory response in AD. We show that inhibition of CK2 reduces part of the inflammatory response driven by astrocytes. Whether CK2 is functionally involved in early phases of Aβ pathology remains elusive and should be investigated in future studies.

Aberrant CK2 signalling has been implicated in several inflammatory diseases such as T cell lymphoma [73], breast cancer [26, 74, 75] and autoimmune encephalomyelitis [76]. CK2 is known to mediate the regulation of key transcription factors and cytokines such as serum IL-6 and the signal transducer and activator of transcription 3 (STAT3) [74] and nuclear factor kappa-light-chain-enhancer of activated B cells (NF-κB). The mechanism of NF-κB regulation by CK2 has been well-studied in epithelial cells [26, 31] and in cell models such as human cancerous HeLa cells [77] and diploid gingival fibroblasts [26, 27]. In turn, TNF-α has been shown to stimulate CK2 activity in Swiss 3T3, L929 and human cervical carcinoma HeLa cells [29, 30], and IL-1 has been found to activate CK2 in intestinal epithelial cells [31]. In addition to increased presence of CK2 positive astrocytes around amyloid plaques, we observed increased presence of CK2 positive astrocytes associated with CAA in AD. The chronically increased production of inflammatory cytokines is one of the causes of blood-brain barrier (BBB) dysfunction [5, 78]. This suggests that CK2 could be involved in the inflammatory driven dysfunction of the BBB observed in AD.

The ATP-competitive CK2 inhibitor TBB has been used widely as a molecular probe to elucidate the functional role of CK2 [38]. In this study, we report that TBB had no significant effect on the IL-1β/TNF-α mediated secretion of IL-6 and MCP-1 by human primary astrocytes and U373 cells. Previously, Kramerov et al. reported a minimal effective concentration of 75 μM TBB for retinal astrocytes in culture [69]. In the current study, we tested concentrations up to 20 μM due to decline of cell viability when using higher concentrations.

The selective CK2 inhibitor CX-4945 on the other hand led to significant inhibition of the IL-1β/TNF-α mediated secretion of IL-6 and MCP-1 by human primary astrocytes and U373 cells. CK2 overexpression has been implicated in a number of different cancers including head and neck [79], colorectal [80], renal [81], lung [82], leukemias [83] and prostate cancer [84]. It has been reported that angiogenesis and proliferation are regulated by CK2 and that CK2 is an essential protein for cancer cell survival [85]. Downregulation of CK2 activity with specific inhibitors, like CX-4945, could reduce cancer cell viability and induce apoptosis [40, 74]. CX-4945 exerts strong anti-proliferative activity and also downregulates signalling cascades that act downstream of BCR, including PI3K/Akt/mTOR signalling by directly blocking the phosphorylation of Akt at Serine 129 by CK2 [40, 85]. CX-4945 is currently the only CK2 inhibitor used in clinical trials for cancer therapy [39, 40]. In phase I clinical trials for patients with different solid tumours, adverse effects of CX-4945 were generally mild to moderate, demonstrating that CX-4945 can be safely administered to humans [41, 85-86]. Chon et al. reported that CX-4945 in combination with other inhibitors yielded synergistic effects in cell death induction making this inhibitor a promising therapeutic tool for the treatment of cancer and possibly other inflammatory diseases such as AD [40].

Overall, our data suggests a role for protein kinase CK2 in the neuroinflammatory response in AD. Although neuroinflammation in the brain of AD patients is considered primarily beneficial, i.e. removing Aβ aggregates from the brain, a chronic neuroinflammatory response is thought to be harmful due to the constant excess production of pro-inflammatory cytokines, prostaglandins and reactive oxygen species that exacerbate Aβ deposition and induce neuronal dysfunction [87]. However, all clinical trials with AD patients using anti-inflammatory drugs have failed so far, indicating the need for new anti-inflammatory treatments [88]. Protein kinases can be targeted by relatively small compounds that are able to pass the BBB [17]. Therefore, the regulation of protein kinase activity by small ligand molecules seems very promising for future drug-based therapy directed at inflammation or neurodegeneration. The anti-inflammatory effects of CX-4945 on human astrocytes suggest that the CK2 signalling pathway could act as a potential therapeutic target for modulating neuroinflammation in AD. Whether CX-4945 is able to pass the BBB in order to reduce neuroinflammation in the brain needs to be resolved.

## Conclusions

In conclusion, we found that CK2α/α’ is increased in astrocytes in the hippocampus and temporal cortex of AD patients. CK2α/α’ immunoreactive astrocytes are associated with amyloid deposits in AD brain. The selective CK2 inhibitor CX-4945 significantly reduced the IL-1β/TNF-α induced secretion of the inflammatory cytokines MCP-1 and IL-6 both in human primary astrocytes and U373 astrocytoma cells in a dose-dependent manner. This suggests that CK2α/α’ is a modulator of neuroinflammation in AD.

## Additional files

Additional file 1: Figure S3. Detection of CK2α/α’ on formalin fixed paraffin embedded tissue. Five-micrometer-thick sections from formalin-fixed paraffin tissue were mounted on superfrost plus tissue slides (Menzel-Gläser, Germany) and dried overnight at 37 °C. Sections were deparaffinised and subsequently immersed in 0.3 % H_2_O_2_ in methanol for 30 min to quench endogenous peroxidase activity. Between the subsequent incubation steps, sections were washed extensively with PBS. Sections were treated in 10 mM pH 6.0 sodium citrate buffer heated by autoclave during 10 min for antigen retrieval. Mouse monoclonal anti-CK2α (1:100, Santa Cruz Biotechnology, CA) was diluted in antibody diluent (Immunologic) and incubated overnight at 4 °C. Omission of the primary antibody served as a negative control. Secondary EnVisonTM HRP goat anti-rabbit/mouse antibody (EV-GαMHRP, Dako) incubation was for 30 min at 4 °C. The secondary antibody was detected using 3,3-diaminobenzidine (Dako). Sections were counterstained with haematoxylin for 1 min, dehydrated and mounted using Quick-D mounting medium (BDH Laboratories Supplies, Poole, England). Shown are representative pictures from the temporal cortex of an AD case with Braak 6 for neurofibrillary tangles. Scale bar A 200 μm, B 50 μm. (PDF 208 kb) Additional file 2: Figure S1. Specificity of CK2α and CK2α’ antibodies. Western blot analysis of recombinant CK2α and CK2α’ in three different concentrations. Incubation with either mouse anti-CK2α or goat anti-CK2α’ goat antibody using a 1:1000 dilution was performed overnight. (PDF 104 kb) Additional file 3: Figure S2. Expression of CK2 in the hippocampus and temporal cortex. Protein expression of CK2 was assessed by Western blot analysis using mouse anti-CK2 which detects both CK2α and CK2α’ (see Additional file 2: Figure S1). a Western blot analysis of brain extracts from the hippocampus. b Western blot analysis of brain extracts from the temporal cortex. AD and non-demented control (CON) cases analysed by Western blotting are listed in (Table 1). Braak stages are indicated, and recombinant CK2α and CK2α’ were used as positive controls. (PDF 137 kb)

## References

[1] Selkoe DJ (2001). Alzheimer’s disease: genes, proteins, and therapy. Physiol Rev.

[2] Carrano A, Hoozemans JJM, Van Der Vies SM, Van Horssen J, De Vries HE, Rozemuller AJM (2012). Neuroinflammation and blood-brain barrier changes in capillary amyloid angiopathy. Neurodegener Dis.

[3] Thal DR, Ghebremedhin E, Rüb U, Yamaguchi H, Del Tredici K, Braak H (2002). Two types of sporadic cerebral amyloid angiopathy. J Neuropathol Exp Neurol.

[4] Hoozemans JJM, Van Haastert ES, Mulder SD, Nielsen HM, Veerhuis R, Ruijtenbeek R (2014). Increased IRAK-4 kinase activity in Alzheimer’s disease; inhibitory effect of IRAK-1/4 inhibitor I on pro-inflammatory cytokine secretion but not on uptake of amyloid beta by human glial cells. J Clin Cell Immunol.

[5] Dong Y, Benveniste EN (2001). Immune function of astrocytes. Glia.

[6] Arends YM, Duyckaerts C, Rozemuller JM, Eikelenboom P, Hauw JJ (2000). Microglia, amyloid and dementia in Alzheimer disease: a correlative study. Neurobiol Aging.

[7] Vehmas AK, Kawas CH, Stewart WF, Troncoso JC (2003). Immune reactive cells in senile plaques and cognitive decline in Alzheimer’s disease. Neurobiol Aging.

[8] Hoozemans JJM, van Haastert ES, Veerhuis R, Arendt T, Scheper W, Eikelenboom P (2005). Maximal COX-2 and ppRb expression in neurons occurs during early Braak stages prior to the maximal activation of astrocytes and microglia in Alzheimer’s disease. J Neuroinflammation.

[9] Lambert J-C, Heath S, Even G, Campion D, Sleegers K, Hiltunen M (2009). Genome-wide association study identifies variants at CLU and CR1 associated with Alzheimer’s disease. Nat Genet.

[10] Harold D, Abraham R, Hollingworth P, Sims R, Gerrish A, Marian L (2009). Genome-wide association study identifies variants at CLU and PICALM associated with Alzheimer’ s disease. Nat Genet.

[11] Naj AC, Jun G, Beecham GW, Wang L-S, Vardarajan BN, Buros J (2011). Common variants at MS4A4/MS4A6E, CD2AP, CD33 and EPHA1 are associated with late-onset Alzheimer’s disease. Nat Genet.

[12] Hollingworth P, Harold D, Sims R, Gerrish A, Lambert J-C, Carrasquillo MM (2011). Common variants at ABCA7, MS4A6A/MS4A4E, EPHA1, CD33 and CD2AP are associated with Alzheimer’s disease. Nat Genet.

[13] Jonsson T, Stefansson H, Steinberg S, Jonsdottir I, Jonsson PV, Snaedal J (2013). Variant of TREM2 associated with the risk of Alzheimer’s disease. N Engl J Med.

[14] Guerreiro R, Wojtas A, Bras J, Carrasquillo M, Rogaeva E, Majounie E (2013). TREM2 variants in Alzheimer’s disease. N Engl J Med.

[15] Glass CK, Saijo K, Winner B, Marchetto MC, Gage FH. Mechanisms underlying inflammation in neurodegeneration. Cell. 2010;918–934. 10.1016/j.cell.2010.02.016 PMC2873093 20303880

[16] Rosenberger AFN, Hilhorst R, Coart E, García Barrado L, Naji F, Rozemuller AJM, et al. Protein kinase activity decreases with higher Braak stages of Alzheimer’s disease pathology. J Alzheimers Dis. 2015. 10.3233/JAD-150429 PMC4927853 26519433

[17] Cohen P (2002). Protein kinases—the major drug targets of the twenty-first century?. Nat Rev Drug Discov.

[18] Iimoto DS, Masliah E, Deteresa R, Terry RD, Saitoh T (1990). Aberrant casein kinase II in Alzheimer’s disease. Brain Res.

[19] Allende JE, Allende CC (1995). Protein kinases. 4. Protein kinase CK2: an enzyme with multiple substrates and a puzzling regulation. FASEB J.

[20] Perez DI, Gil C, Martinez A (2011). Protein kinases CK1 and CK2 as new targets for neurodegenerative diseases. Med Res Rev.

[21] Kramerov AA, Saghizadeh M, Pan H, Kabosova A, Montenarh M, Ahmed K (2006). Expression of protein kinase CK2 in astroglial cells of normal and neovascularized retina. Am J Pathol.

[22] Shi X, Potvin B, Huang T, Hilgard P, Spray DC, Suadicani SO (2001). A novel casein kinase 2 alpha-subunit regulates membrane protein traffic in the human hepatoma cell line HuH-7. J Biol Chem.

[23] Meggio F, Pinna LA (2003). One-thousand-and-one substrates of protein kinase CK2?. FASEB J.

[24] Litchfield DW (2003). Protein kinase CK2: structure, regulation and role in cellular decisions of life and death. Biochem J.

[25] Hériché JK, Chambaz EM (1998). Protein kinase CK2alpha is a target for the Abl and Bcr-Abl tyrosine kinases. Oncogene.

[26] Singh NN, Ramji DP (2008). Protein kinase CK2, an important regulator of the inflammatory response?. J Mol Med (Berl).

[27] Bird TA, Schooley K, Dower SK, Hagen H, Virca GD (1997). Activation of nuclear transcription factor NF-κB by interleukin-1 is accompanied by casein kinase II-mediated phosphorylation of the p65 subunit. J Biol Chem.

[28] Lodie TA, Savedra R, Golenbock DT, Van Beveren CP, Maki RA, Fenton MJ (1997). Stimulation of macrophages by lipopolysaccharide alters the phosphorylation state, conformation, and function of PU.1 via activation of casein kinase II. J Immunol.

[29] Van Lint J, Agostinis P, Vandevoorde V, Haegeman G, Fiers W, Merlevede W (1992). Tumor necrosis factor stimulates multiple serine/threonine protein kinases in Swiss 3 T3 and L929 cells. Implication of casein kinase-2 and extracellular signal-regulated kinases in the tumor necrosis factor signal transduction pathway. J Biol Chem.

[30] Sayed M, Kim SO, Salh BS, Issinger OG, Pelech SL (2000). Stress-induced activation of protein kinase CK2 by direct interaction with p38 mitogen-activated protein kinase. J Biol Chem.

[31] Parhar K, Morse J, Salh B (2007). The role of protein kinase CK2 in intestinal epithelial cell inflammatory signaling. Int J Colorectal Dis.

[32] Singh NN, Ramji DP (2006). Transforming growth factor-beta-induced expression of the apolipoprotein E gene requires c-Jun N-terminal kinase, p38 kinase, and casein kinase 2. Arterioscler Thromb Vasc Biol.

[33] Zdunek M, Silbiger S, Lei J, Neugarten J (2001). Protein kinase CK2 mediates TGF-beta1-stimulated type IV collagen gene transcription and its reversal by estradiol. Kidney Int.

[34] Mead JR, Hughes TR, Irvine SA, Singh NN, Ramji DP (2003). Interferon-gamma stimulates the expression of the inducible cAMP early repressor in macrophages through the activation of casein kinase 2: a potentially novel pathway for interferon mediated inhibition of gene transcription. J Biol Chem.

[35] Harvey EJ, Li N, Ramji DP (2007). Critical role for casein kinase 2 and phosphoinositide-3-kinase in the interferon-gamma-induced expression of monocyte chemoattractant protein-1 and other key genes implicated in atherosclerosis. Arterioscler Thromb Vasc Biol.

[36] Duncan JS, Litchfield DW (2008). Too much of a good thing: the role of protein kinase CK2 in tumorigenesis and prospects for therapeutic inhibition of CK2. Biochim Biophys Acta.

[37] Sarno S, Ruzzene M, Frascella P, Pagano MA, Meggio F, Zambon A (2005). Development and exploitation of CK2 inhibitors. Mol Cell Biochem.

[38] Sarno S, Reddy H, Meggio F, Ruzzene M, Davies SP, Donella-Deana A (2001). Selectivity of 4,5,6,7-tetrabromobenzotriazole, an ATP site-directed inhibitor of protein kinase CK2 (‘casein kinase-2’). FEBS Lett.

[39] Pierre F, Chua PC, O’Brien SE, Siddiqui-Jain A, Bourbon P, Haddach M (2011). Discovery and SAR of 5-(3-chlorophenylamino)benzo[c][2,6]naphthyridine-8-carboxylic acid (CX-4945), the first clinical stage inhibitor of protein kinase CK2 for the treatment of cancer. J Med Chem.

[40] Chon HJ, Bae KJ, Lee Y, Kim J (2015). The casein kinase 2 inhibitor, CX-4945, as an anti-cancer drug in treatment of human hematological malignancies. Front Pharmacol.

[41] Martins LR, Lúcio P, Melão A, Antunes I, Cardoso BA, Stansfield R (2014). Activity of the clinical-stage CK2-specific inhibitor CX-4945 against chronic lymphocytic leukemia. Leukemia.

[42] Braak H, Alafuzoff I, Arzberger T, Kretzschmar H, Del Tredici K (2006). Staging of Alzheimer disease-associated neurofibrillary pathology using paraffin sections and immunocytochemistry. Acta Neuropathol.

[43] Thal DR, Rüb U, Schultz C, Sassin I, Ghebremedhin E, Del Tredici K (2000). Sequence of Abeta-protein deposition in the human medial temporal lobe. J Neuropathol Exp Neurol.

[44] Thal DR, Walter J, Saido TC, Fändrich M (2015). Neuropathology and biochemistry of Aβ and its aggregates in Alzheimer’s disease. Acta Neuropathol.

[45] Reisberg B, Ferris SH, De Leon MJ, Crook T (1982). The global deterioration scale for assessment of primary degenerative dementia. Am J Psychiatry.

[46] Hyman BT, Phelps CH, Beach TG, Bigio EH, Cairns NJ, Carrillo MC (2012). National Institute on Aging-Alzheimer’s Association guidelines for the neuropathologic assessment of Alzheimer’s disease. Alzheimers Dement.

[47] Crary JF, Trojanowski JQ, Schneider JA, Abisambra JF, Abner EL, Alafuzoff I (2014). Primary age-related tauopathy (PART): a common pathology associated with human aging. Acta Neuropathol.

[48] Duyckaerts C, Braak H, Brion J-P, Buée L, Del Tredici K, Goedert M (2015). PART is part of Alzheimer disease. Acta Neuropathol.

[49] Spillantini MG, Crowther RA, Goedert M (1996). Comparison of the neurofibrillary pathology in Alzheimer’s disease and familial presenile dementia with tangles. Acta Neuropathol.

[50] de Groot CJ, Hulshof S, Hoozemans JJ, Veerhuis R (2001). Establishment of microglial cell cultures derived from postmortem human adult brain tissue: immunophenotypical and functional characterization. Microsc Res Tech.

[51] Hoozemans JJM, Veerhuis R, Janssen I, van Elk E-J, Rozemuller AJM, Eikelenboom P (2002). The role of cyclo-oxygenase 1 and 2 activity in prostaglandin E(2) secretion by cultured human adult microglia: implications for Alzheimer’s disease. Brain Res.

[52] Alley MC, Scudiero DA, Monks A, Hursey Czerwinski MLMJ, Fine DL, Abbott BJ (1988). Feasibility of drug screening with panels of human tumor cell lines using a microculture tetrazolium assay. Cancer Res.

[53] Ruzzene M, Penzo D, Pinna LA (2002). Protein kinase CK2 inhibitor 4,5,6,7-tetrabromobenzotriazole (TBB) induces apoptosis and caspase-dependent degradation of haematopoietic lineage cell-specific protein 1 (HS1) in Jurkat cells. Biochem J.

[54] Lieb K, Fiebich BL, Hüll M, Berger M, Bauer J (1997). Potent inhibition of interleukin-6 expression in a human astrocytoma cell line by tenidap. Cell Tissue Res.

[55] Blom MA, van Twillert MG, de Vries SC, Engels F, Finch CE, Veerhuis R (1997). NSAIDS inhibit the IL-1 beta-induced IL-6 release from human post-mortem astrocytes: the involvement of prostaglandin E2. Brain Res.

[56] Blanquet PR. Casein kinase 2 as a potentially important enzyme in the nervous system. Prog Neurobiol. 2000;211–246. 10.1016/s0301-0082(99)00026-x 10658642

[57] Faust M, Montenarh M (2000). Subcellular localization of protein kinase CK2. Cell Tissue Res.

[58] Chung HJ, Huang YH, Lau L-F, Huganir RL (2004). Regulation of the NMDA receptor complex and trafficking by activity-dependent phosphorylation of the NR2B subunit PDZ ligand. J Neurosci.

[59] Kimura R, Matsuki N (2008). Protein kinase CK2 modulates synaptic plasticity by modification of synaptic NMDA receptors in the hippocampus. J Physiol.

[60] Lenzken SC, Stanga S, Lanni C, De Leonardis F, Govoni S, Racchi M (2010). Recruitment of casein kinase 2 is involved in AbetaPP processing following cholinergic stimulation. J Alzheimers Dis.

[61] Raftery M, Campbell R, Glaros EN, Rye K-A, Halliday GM, Jessup W (2005). Phosphorylation of apolipoprotein-E at an atypical protein kinase CK2 PSD/E site in vitro. Biochemistry.

[62] Walter J, Schindzielorz A, Hartung B, Haass C (2000). Phosphorylation of the beta-amyloid precursor protein at the cell surface by ectocasein kinases 1 and 2. J Biol Chem.

[63] Baum L, Masliah E, Iimoto DS, Hansen LA, Halliday WC, Saitoh T (1992). Casein kinase II is associated with neurofibrillary tangles but is not an intrinsic component of paired helical filaments. Brain Res.

[64] Lim ACB, Tiu S-Y, Li Q, Qi RZ (2004). Direct regulation of microtubule dynamics by protein kinase CK2. J Biol Chem.

[65] De Felice FG, Vieira MNN, Bomfim TR, Decker H, Velasco PT, Lambert MP (2009). Protection of synapses against Alzheimer’s-linked toxins: insulin signaling prevents the pathogenic binding of Abeta oligomers. Proc Natl Acad Sci U S A.

[66] Aksenova MV, Burbaeva GS, Kandror KV, Kapkov DV, Stepanov AS (1991). The decreased level of casein kinase 2 in brain cortex of schizophrenic and Alzheimer’s disease patients. FEBS Lett.

[67] Saitoh T, Iimoto D (1989). Aberrant protein phosphorylation and cytoarchitecture in Alzheimer’s disease. Prog Clin Biol Res.

[68] Masliah E, Iimoto DS, Mallory M, Albright T, Hansen L, Saitoh T (1992). Casein kinase II alteration precedes tau accumulation in tangle formation. Am J Pathol.

[69] Kramerov AA, Golub AG, Bdzhola VG, Yarmoluk SM, Ahmed K, Bretner M (2011). Treatment of cultured human astrocytes and vascular endothelial cells with protein kinase CK2 inhibitors induces early changes in cell shape and cytoskeleton. Mol Cell Biochem.

[70] Kramerov AA, Ahmed K, Ljubimov AV (2012). Cell rounding in cultured human astrocytes and vascular endothelial cells upon inhibition of CK2 is mediated by actomyosin cytoskeleton alterations. J Cell Biochem.

[71] Nielsen HM, Veerhuis R, Holmqvist B, Janciauskiene S (2009). Binding and uptake of A beta1-42 by primary human astrocytes in vitro. Glia.

[72] Thal DR, Schultz C, Dehghani F, Yamaguchi H, Braak H, Braak E (2000). Amyloid beta-protein (Abeta)-containing astrocytes are located preferentially near N-terminal-truncated Abeta deposits in the human entorhinal cortex. Acta Neuropathol.

[73] Seldin DC, Leder P (1995). Casein kinase II alpha transgene-induced murine lymphoma: relation to theileriosis in cattle. Science.

[74] Drygin D, Ho CB, Omori M, Bliesath J, Proffitt C, Rice R (2011). Protein kinase CK2 modulates IL-6 expression in inflammatory breast cancer. Biochem Biophys Res Commun.

[75] Landesman-Bollag E, Song DH, Romieu-Mourez R, Sussman DJ, Cardiff RD, Sonenshein GE (2001). Protein kinase CK2: signaling and tumorigenesis in the mammary gland. Mol Cell Biochem.

[76] Axtell RC, Xu L, Barnum SR, Raman C (2006). CD5-CK2 binding/activation-deficient mice are resistant to experimental autoimmune encephalomyelitis: protection is associated with diminished populations of IL-17-expressing T cells in the central nervous system. J Immunol.

[77] Wang D, Westerheide SD, Hanson JL, Baldwin AS (2000). Tumor necrosis factor alpha-induced phosphorylation of RelA/p65 on Ser529 is controlled by casein kinase II. J Biol Chem.

[78] Zhang F, Jiang L (2015). Neuroinflammation in Alzheimer’s disease. Neuropsychiatr Dis Treat.

[79] Faust RA, Tawfic S, Davis AT, Bubash LA, Ahmed K (2000). Antisense oligonucleotides against protein kinase CK2-alpha inhibit growth of squamous cell carcinoma of the head and neck in vitro. Head Neck.

[80] Pistorius K, Seitz G, Remberger K, Issinger OG (1991). Differential CKII activities in human colorectal mucosa, adenomas and carcinomas. Onkologie.

[81] Stalter G, Siemer S, Becht E, Ziegler M, Remberger K, Issinger OG (1994). Asymmetric expression of protein kinase CK2 subunits in human kidney tumors. Biochem Biophys Res Commun.

[82] O-charoenrat P, Rusch V, Talbot SG, Sarkaria I, Viale A, Socci N (2004). Casein kinase II alpha subunit and C1-inhibitor are independent predictors of outcome in patients with squamous cell carcinoma of the lung. Clin Cancer Res.

[83] Kim JS, Eom JI, Cheong J-W, Choi AJ, Lee JK, Yang WI (2007). Protein kinase CK2alpha as an unfavorable prognostic marker and novel therapeutic target in acute myeloid leukemia. Clin Cancer Res.

[84] Yenice S, Davis AT, Goueli SA, Akdas A, Limas C, Ahmed K (1994). Nuclear casein kinase 2 (CK-2) activity in human normal, benign hyperplastic, and cancerous prostate. Prostate.

[85] Siddiqui-Jain A, Drygin D, Streiner N, Chua P, Pierre F, O'Brien SE (2010). CX-4945, an orally bioavailable selective inhibitor of protein kinase CK2, inhibits prosurvival and angiogenic signaling and exhibits antitumor efficacy. Cancer Res.

[86] Cozza G, Pinna LA, Moro S (2013). Kinase CK2 inhibition: an update. Curr Med Chem.

[87] Wyss-Coray T, Mucke L (2002). Inflammation in neurodegenerative disease—a double-edged sword. Neuron.

[88] van Gool WA, Aisen PS, Eikelenboom P (2003). Anti-inflammatory therapy in Alzheimer’s disease: is hope still alive?. J Neurol.

