# Supplementary figures and images for: Increased occurrence of protein kinase CK2 in astrocytes in Alzheimer’s disease pathology
Source: J Neuroinflammation. 2016 Jan 6;13:4. doi: 10.1186/s12974-015-0470-x (PMC4702323; doi:10.1186/s12974-015-0470-x)

Figure S3

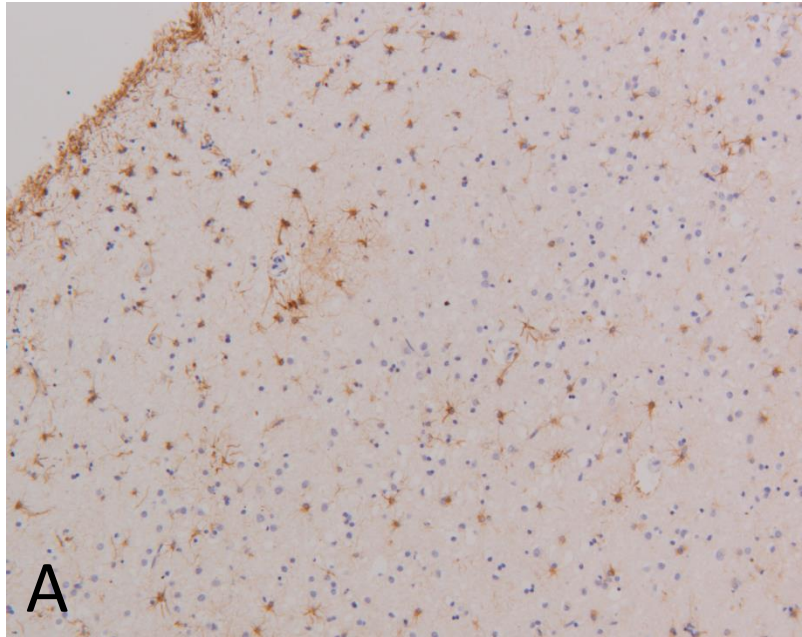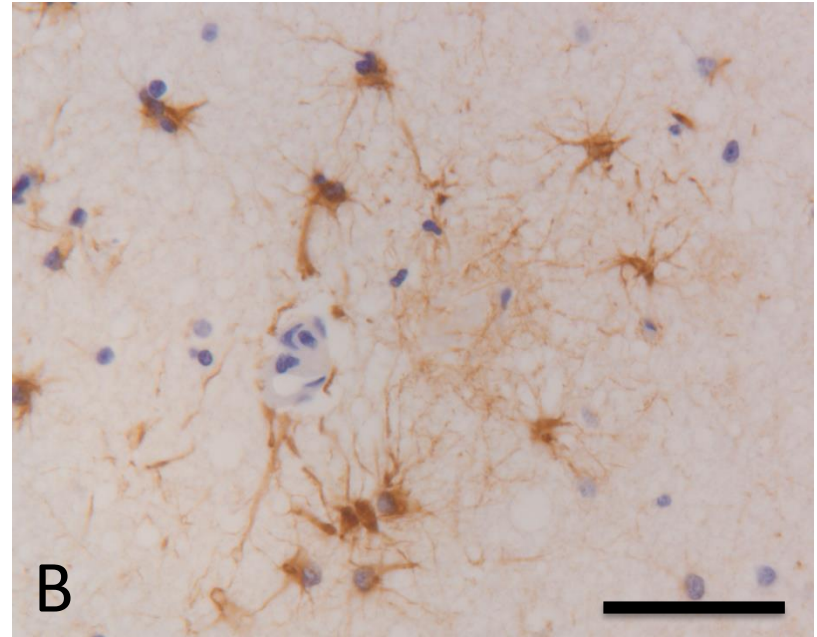

Supplement: Additional file 1: Figure S3. — Detection of CK2α/α’ on formalin fixed paraffin embedded tissue. Five-micrometer-thick sections from formalin-fixed paraffin tissue were mounted on superfrost plus tissue slides (Menzel-Gläser, Germany) and dried overnight at 37 °C. Sections were deparaffinised and subsequently immersed in 0.3 % H2O2 in methanol for 30 min to quench endogenous peroxidase activity. Between the subsequent incubation steps, sections were washed extensively with PBS. Sections were treated in 10 mM pH 6.0 sodium citrate buffer heated by autoclave during 10 min for antigen retrieval. Mouse monoclonal anti-CK2α (1:100, Santa Cruz Biotechnology, CA) was diluted in antibody diluent (Immunologic) and incubated overnight at 4 °C. Omission of the primary antibody served as a negative control. Secondary EnVisonTM HRP goat anti-rabbit/mouse antibody (EV-GαMHRP, Dako) incubation was for 30 min at 4 °C. The secondary antibody was detected using 3,3-diaminobenzidine (Dako). Sections were counterstained with haematoxylin for 1 min, dehydrated and mounted using Quick-D mounting medium (BDH Laboratories Supplies, Poole, England). Shown are representative pictures from the temporal cortex of an AD case with Braak 6 for neurofibrillary tangles. Scale bar A 200 μm, B 50 μm. (PDF 208 kb) [file 12974_2015_470_MOESM1_ESM.pdf]

# Figure S1

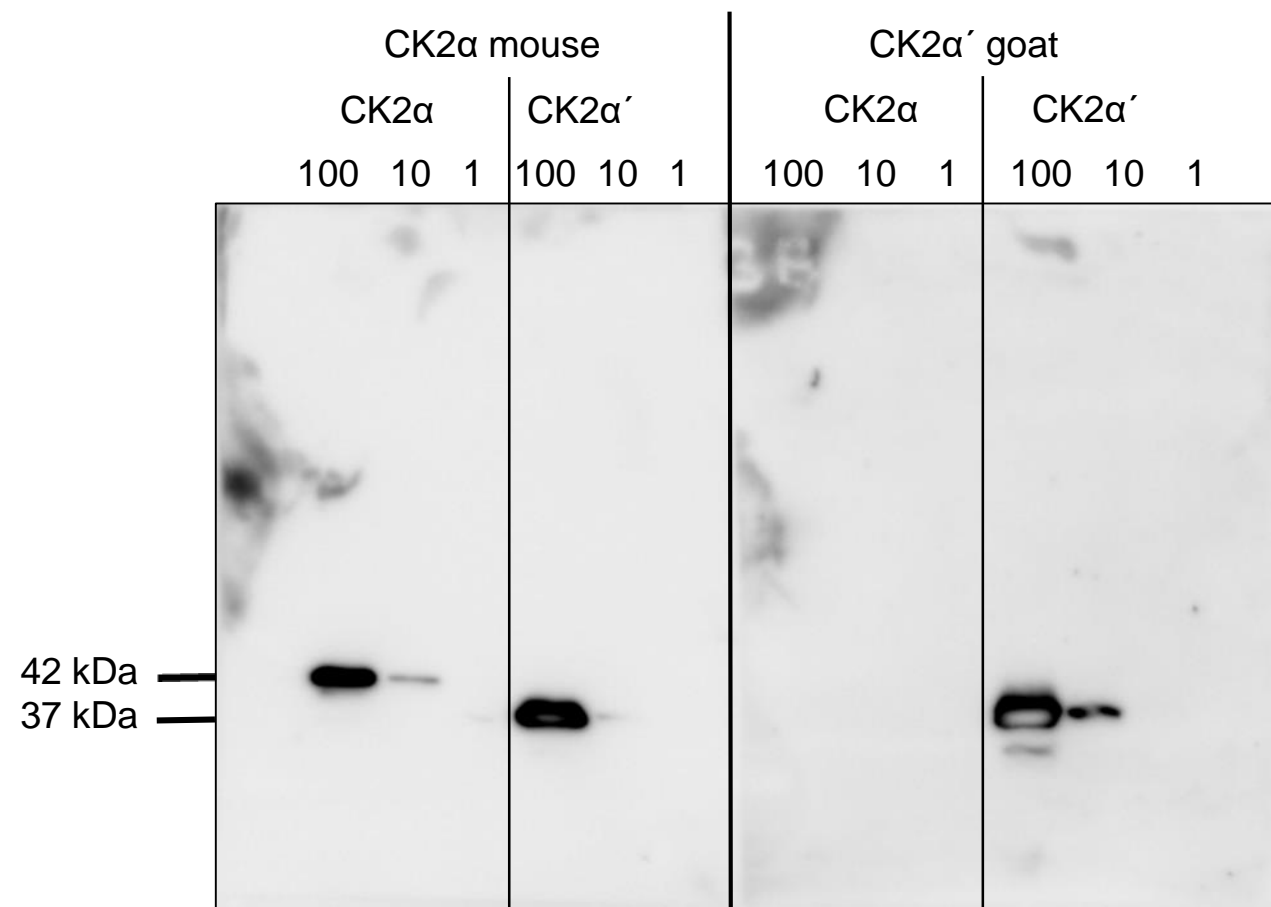

Supplement: Additional file 2: Figure S1. — Specificity of CK2α and CK2α’ antibodies. Western blot analysis of recombinant CK2α and CK2α’ in three different concentrations. Incubation with either mouse anti-CK2α or goat anti-CK2α’ goat antibody using a 1:1000 dilution was performed overnight. (PDF 104 kb) [file 12974_2015_470_MOESM2_ESM.pdf]

Figure S2

**A**

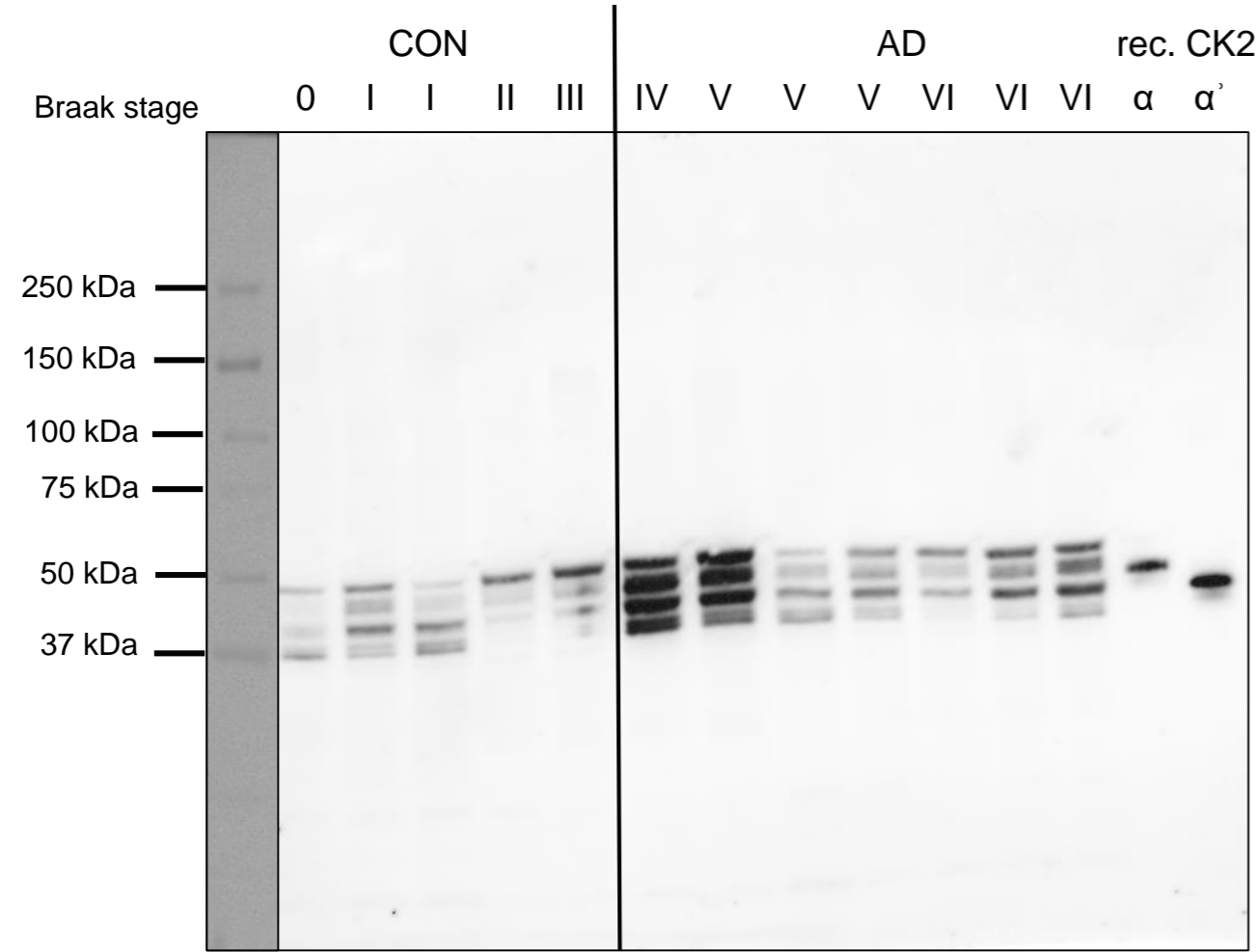

**B**

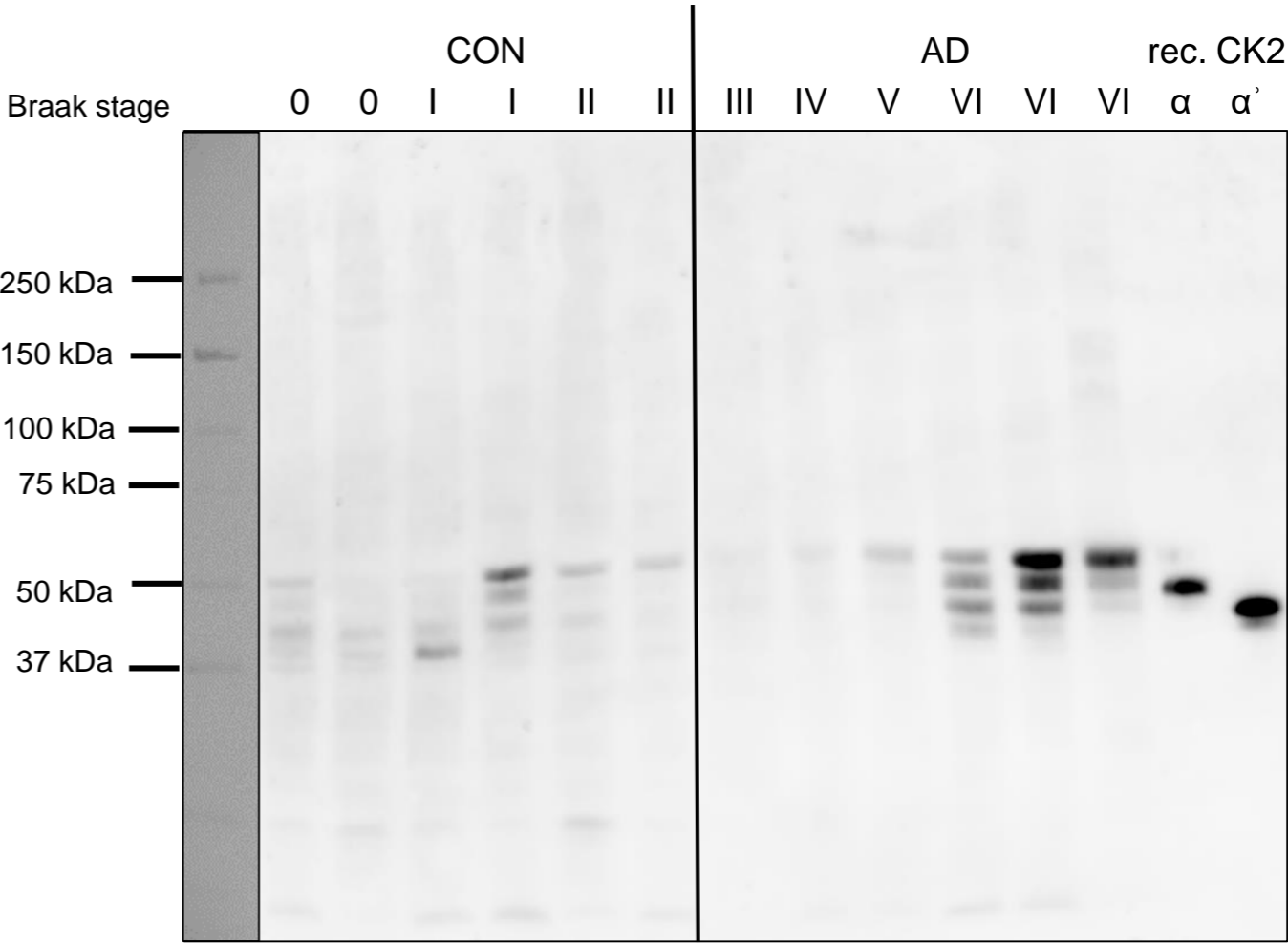

Supplement: Additional file 3: Figure S2. — Expression of CK2 in the hippocampus and temporal cortex. Protein expression of CK2 was assessed by Western blot analysis using mouse anti-CK2 which detects both CK2α and CK2α’ (see Additional file 2: Figure S1). a Western blot analysis of brain extracts from the hippocampus. b Western blot analysis of brain extracts from the temporal cortex. AD and non-demented control (CON) cases analysed by Western blotting are listed in (Table 1). Braak stages are indicated, and recombinant CK2α and CK2α’ were used as positive controls. (PDF 137 kb) [file 12974_2015_470_MOESM3_ESM.pdf]
